# Alternative Splicing of *Cdh23* Exon 68 Is Regulated by RBM24, RBM38, and PTBP1

**DOI:** 10.1155/2020/8898811

**Published:** 2020-07-25

**Authors:** Nana Li, Haibo Du, Rui Ren, Yanfei Wang, Zhigang Xu

**Affiliations:** ^1^Shandong Provincial Key Laboratory of Animal Cell and Developmental Biology, School of Life Sciences, Shandong University, Qingdao, Shandong Province, China; ^2^Shandong Provincial Collaborative Innovation Center of Cell Biology, Shandong Normal University, Jinan, Shandong Province, China

## Abstract

Alternative splicing plays a pivotal role in modulating the function of eukaryotic proteins. In the inner ear, many genes undergo alternative splicing, and errors in this process lead to hearing loss. Cadherin 23 (CDH23) forms part of the so-called tip links, which are indispensable for mechanoelectrical transduction (MET) in the hair cells. *Cdh23* gene contains 69 exons, and exon 68 is subjected to alternative splicing. Exon 68 of the *Cdh23* gene is spliced into its mRNA only in a few cell types including hair cells. The mechanism responsible for the alternative splicing of *Cdh23* exon 68 remains elusive. In the present work, we performed a cell-based screening to look for splicing factors that regulate the splicing of *Cdh23* exon 68. RBM24 and RBM38 were identified to enhance the inclusion of *Cdh23* exon 68. The splicing of *Cdh23* exon 68 is affected in *Rbm24* knockdown or knockout cells. Moreover, we also found that PTBP1 inhibits the inclusion of *Cdh23* exon 68. Taken together, we show here that alternative splicing of *Cdh23* exon 68 is regulated by RBM24, RBM38, and PTBP1.

## 1. Introduction

In eukaryotic cells, the exons of precursor mRNAs (pre-mRNAs) are spliced together by a large protein complex named spliceosome to give rise to mature mRNAs [1]. Many genes undergo alternative splicing, which results in distinct mature mRNAs through splicing different exons together [2]. In this way, alternative splicing helps to produce structurally and functionally similar but not identical protein isoforms, hence contributing to proteomic diversity [3]. Tissue-specific alternative splicing also contributes to tissue specificity. Alternative splicing is regulated by specific RNA sequences and related RNA-binding proteins [2]. The RNA sequences that regulate alternative splicing are divided into different classes according to their position and function, namely, exonic splicing enhancers (ESEs), exonic splicing silencers (ESSs), intronic splicing enhancers (ISEs), and intronic splicing silencers (ISSs), which are bound by different splicing regulators [2]. Tissue-specific alternative splicing is thought to be regulated mainly by differentially expressed splicing regulators [4, 5].

In the inner ear, many important genes have been shown to undergo alternative splicing, and dysregulation of this process causes syndromic or nonsyndromic hearing loss [6]. Cadherin 23 (CDH23) is an atypical cadherin, forming part of the so-called tip links that play a pivotal role in mechanoelectrical transduction (MET) in the hair cells [7–10]. Mouse *Cdh23* gene contains 69 exons, and exon 68 is subjected to alternative splicing [11]. Interestingly, the inclusion of *Cdh23* exon 68 is only detected in the inner ear so far [8, 12, 13]. *Cdh23* exon 68 is 105 base pair (bp) long, which encodes 35 amino acids (aa) in the cytoplasmic domain of CDH23. The exon 68-encoded peptide shows no homology to any known proteins and might affect the conformation as well as protein-protein interactions of CDH23 [12–16]. At present, the mechanism responsible for the inner ear-specific alternative splicing of *Cdh23* exon 68 is still unknown.

In the present work, we performed a cell-based screening in order to identify the splicing factors that regulate the alternative splicing of *Cdh23* exon 68. The screening identified RBM24 and RBM38 that enhance the inclusion of *Cdh23* exon 68. Moreover, we also identified PTBP1 that inhibits the inclusion of *Cdh23* exon 68 through bioinformatics analysis. Our data suggest that RBM24, RBM38, and PTBP1 together regulate the alternative splicing of *Cdh23* exon 68.

## 2. Materials and Methods

### 2.1. Reverse Transcription-Polymerase Chain Reaction (RT-PCR)

Total RNA from different mouse tissues or cultured cells was extracted using TRIzol reagent (Invitrogen) according to the manufacturer's protocol. Briefly, 1 *μ*g RNA was used for reverse transcription (RT) using PrimeScript RT Reagent Kit with gDNA Eraser (Takara, RR047A). Polymerase chain reaction (PCR) was then performed using the cDNA as template with the following primers: mouse *Esrp1* forward 5′-CAATATTGCCAAGGGTGGCG-3′, reverse 5′-CTCATGCGCACGATGACTTG-3′; mouse *Rbm24* forward 5′-GAGACATCGAGGAAGCGGTG-3′, reverse 5′-TCTGGATAAGGGCTGGGTGA-3′; mouse *Rbm38* forward 5′-ATGGCAGATCGGGCAGC-3′, reverse 5′-AGCCCGTCTGTAAGCTCCTA-3′; mouse *Ptbp1* forward 5′-AGTGCGCATTACACTGTCCA-3′, reverse 5′-CTTGAGGTCGTCCTCTGACA-3′; mouse *Ptbp2* forward 5′-GCAGAAGAGGATCTGCGAAC-3′, reverse 5′-CATCTTCATCTCCCGTGCTT-3′; mouse *β-actin* forward 5′-CGTTGACATCCGTAAAGACC-3′, reverse 5′-AACAGTCCGCCTAGAAGCAC-3′; mouse *Cdh23* forward 5′-GACAACATCGCCAAGCTG-3′, reverse (for minigene) 5′-GGCCAGCAGTGTGCC-3′; reverse (for endogenous *Cdh23*) 5′-GCAAGCTGTTGAGATCAGTGG-3′; and rat *Cdh23* forward 5′-TGGAACTTTTGGACGTGAGC-3′, reverse 5′-GCTTGTGTCGGAAACGGAGG-3′. For different PCR reactions, 23-40 cycles were used and the annealing temperatures were adjusted between 56 and 64°C to obtain the optimal sensitivity and specificity.

### 2.2. DNA Constructs

The genomic DNA ranging from exon 67 to exon 69 of mouse *Cdh23* gene was PCR amplified and inserted into pmCherry-N1 vector to construct *Cdh23* minigene reporter. Mutation or deletion was introduced into the minigene through overlapping PCR-mediated site-directed mutagenesis. The cDNAs encoding various mouse splicing factors were inserted into pEGFP-C2 or pEGFP-N2 to express GFP-fusion proteins.

### 2.3. Splicing Factor Screening

COS7 cells were transfected with the *Cdh23* minigene reporter and vectors expressing different splicing factors using LipoMax (Sudgen) in 24-well plates. Forty-eight hours after transfection, cells were washed with PBS and fixed with 4% paraformaldehyde (PFA) in PBS for 15 minutes. Florescence was detected using a confocal microscope (LSM 700, Zeiss). ImageJ software was used to quantify the intensity of fluorescence.

### 2.4. RNA Interference and Quantitative Real-Time PCR (qPCR)

Chemically synthesized siRNAs were obtained from Sigma-Aldrich, and their sequences are as follows: siRNA-1, 5′-GCAAUAUGUAGCUUGAAUUdTdT-3′; siRNA-2, 5′-CUUAAGGCCUAUAGAACUUdTdT-3′; siRNA-3, 5′-CCCAAAGAGCCUGAGUAAAdTdT-3′; and control siRNA, 5′-UUCUCCGAACGUGUCACGUTT-3′. COS7 cells were transfected with siRNAs using jetPRIME transfection reagent (Polyplus) in 12-well plates. Twenty-four hours after transfection, the *Cdh23* minigene reporter was transfected into the same cells using LipoMax transfection reagents. Cells were cultured for another twenty-four hours, and RNA was extracted and reversed transcribed as described above. qPCR was then performed with SYBR Premix Ex Taq (Takara, RR420A) and the QuantGene 9600 real-time system (Bioer). Sequences of primers used in the qPCR reactions are as follows: monkey *Rbm24* forward 5′-AGATCGAGGAGGCGGTGGTC-3′, reverse 5′-TCTTTGTATAAGGGCTGGATGAAG-3′; mouse *Cdh23(+68)* forward 5′-AACTCTTTGCACAACGGATG-3′, reverse 5′-ATGGGTGGCTTGTGTCGG-3′; and monkey *β-actin* forward 5′-CGTGGACATCCGCAAAGACC-3′, reverse 5′-CACAGTCCGCCTAGAAGCA-3′.

### 2.5. RNA Immunoprecipitation (RIP)

RIP analysis was carried out according to the standard procedure with modifications [17]. Briefly, COS7 cells were transfected with expression vectors of Myc-tagged splicing factors as well as the *Cdh23* minigene. Forty-eight hours after transfection, cells were lysed in lysis buffer containing 100 mM KCl, 5 mM MgCl_2_, 10 mM HEPES-NaOH, 0.5% NP-40, 1 mM DTT, 200 units/ml RNase inhibitor (Takara, 2313A), and EDTA-free protease inhibitor cocktail (Sigma, S8830). After centrifugation at 4°C, the supernatant was collected and incubated with immobilized anti-Myc antibody (Sigma-Aldrich, Cat. No. E6654) at 4°C for 3 hours. The immunoprecipitated RNA was then used as template for RT-PCR and qPCR analysis. Primers used are as follows: mouse *Cdh23* pre-mRNA forward 5′-CAGCAGCCTAAGTGGGAAG-3′ and reverse 5 ′-AGCTCCCGAGGCTACCTC-3 ′.

### 2.6. Western Blot

COS7 cells were transfected with expression vectors in 6-well plates. Forty-eight hours after transfection, cells were washed with PBS and lysed in ice-cold lysis buffer containing 150 mM NaCl, 50 mM Tris at pH 7.5, 1% Triton X-100, and 1 mM PMSF. After centrifugation at 4°C, the supernatant was collected and separated by 10% polyacrylamide gel electrophoresis (PAGE), then transferred to PVDF membrane. The membrane was blocked in 5% nonfat milk for an hour at room temperature, then incubated with anti-GFP antibody (ABclonal, AE012) at 4°C overnight, followed by incubation with HRP-conjugated secondary antibody (Bio-Rad, 170-6516) for an hour at room temperature. The signals were detected with the ECL system (Cell Signaling Technology).

### 2.7. Animals

All animal experiments were approved by the Ethics Committee of Shandong University School of Life Sciences and conducted accordingly. Construction of *Atoh1-cre*; *Rbm24^flox/flox^* mice is described elsewhere.

### 2.8. Statistical Analysis

Each experiment was repeated at least three times. Student's *t*-test was used to determine the statistical significance, and *p* < 0.05 was considered statistically significant. For alternative splicing events, data were shown as means ± SEM.

## 3. Results

### 3.1. Screening Splicing Factors That Regulate the Alternative Splicing of Cdh23 Exon 68

**Figure 1:**
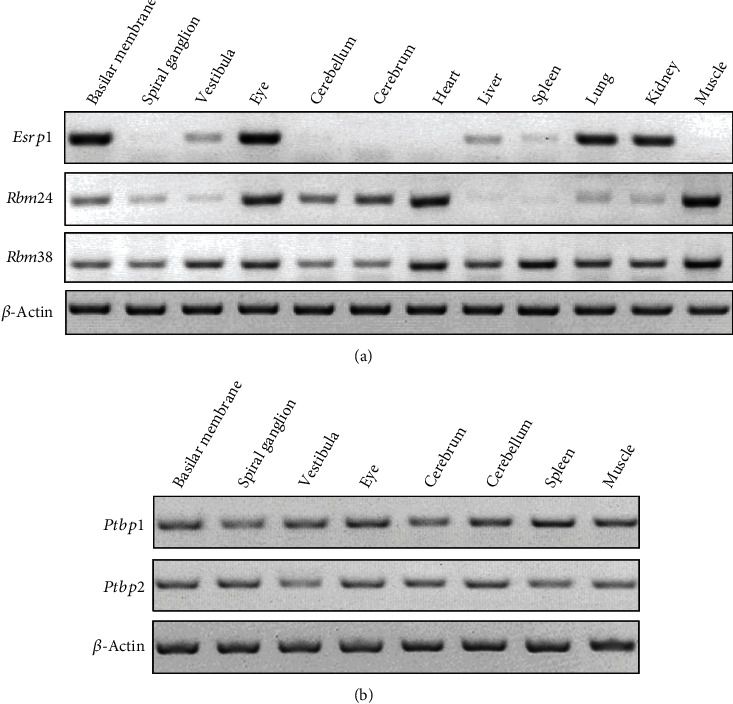
Expression of various splicing regulators in different mouse tissues. RT-PCR analysis was performed to examine the expression of splicing regulators in different tissues of P10 C57BL/6 mice. (a) Expression of *Esrp1*, *Rbm24*, and *Rbm38* was examined by RT-PCR. (b) Expression of *Ptbp1* and *Ptbp2* was examined by RT-PCR. *β*-*Actin* was used as internal control.

To identify splicing factors that regulate the inner ear-specific alternative splicing of *Cdh23* exon 68, we started with testing the known splicing factors that are expressed in the inner ear. Splicing factors SRRM4, SFSWAP, and ESRP1 play important roles in the inner ear, albeit none of them has been shown to regulate the alternative splicing of *Cdh23* exon 68 [18–20]. We previously showed that deafness-related protein ILDR1 and its homolog ILDR2 regulate alternative splicing through binding to splicing factors TRA2A, TRA2B, and SRSF1 [21]. We also found that RNA-binding protein RBM24 is specifically expressed in the hair cells [22]. The expression of these splicing factors in the inner ear is confirmed by performing RT-PCR (Figure 1(a), data not shown). These splicing factors are used as the candidates for the following screening.

**Figure 2:**
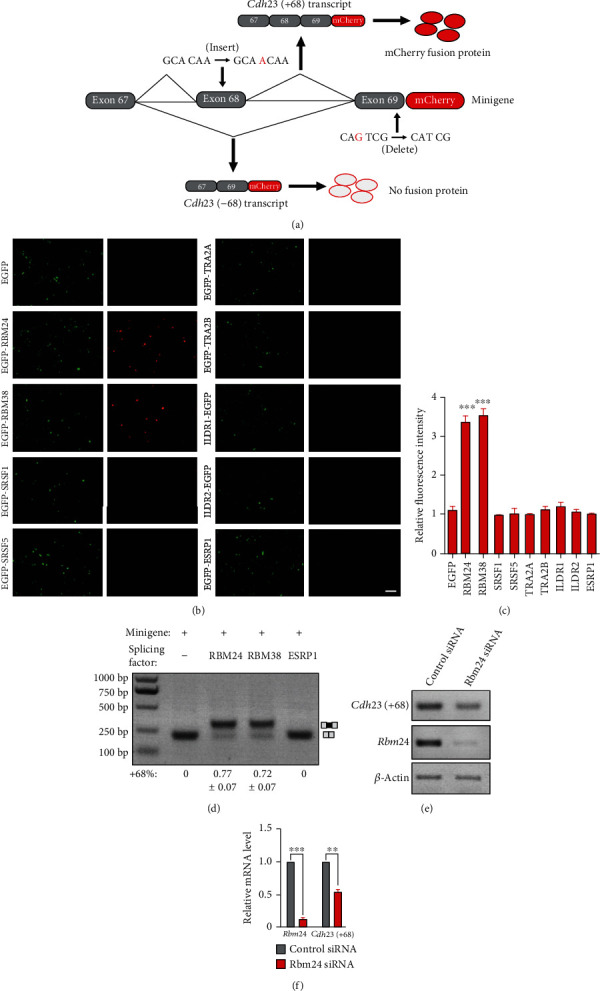
Screening splicing factors that regulate *Cdh23* exon68 inclusion. (a) Schematic drawing of the screening strategy. The *Cdh23* minigene reporter was constructed by fusing mouse *Cdh23* genomic sequence ranging from exon 67 to exon 69 in frame with a mCherry-encoding sequence. A single base pair insertion in exon 68 and a single base pair deletion in exon 69 introduced in the minigene are indicated in red. When exon 68 is included in the mature mRNA, a mCherry fusion protein is expressed. In contrast, when exon 68 is not included in the mature mRNA, the single base pair deletion in exon 69 will cause a frameshift and mCherry fusion protein will not be expressed. (b) The *Cdh23* minigene and various splicing factors were overexpressed in COS7 cells, and the resultant fluorescence was examined using a confocal microscope. Scale bar, 200 *μ*m. (c) The relative mCherry fluorescence intensity from (b) was analyzed using ImageJ software. (d) The *Cdh23* minigene and various splicing factors were overexpressed in COS7 cells, and RT-PCR was performed to examine the inclusion of exon 68. (e, f) The *Cdh23* minigene and either *Rbm24* or control siRNAs were transfected into COS7 cells, and the expression level of *Rbm24* and *Cdh23(+68)* was examined by performing RT-PCR (e) and qPCR (f). *β*-*Actin* was used as internal control. ^∗^*p* < 0.05, ^∗∗^*p* < 0.01, and ^∗∗∗^*p* < 0.001.

A *Cdh23* minigene reporter was constructed by fusing mouse *Cdh23* genomic sequence ranging from exon 67 to exon 69 in frame with a mCherry-encoding sequence whose expression is driven by a CMV promoter (Figure 2(a)). To facilitate the screening, we introduced a single base pair insertion in exon 68 and a single base pair deletion in exon 69. When exon 68 is included in the mature mRNA, the insertion and deletion cancel each other out and a mCherry fusion protein is expressed. However, when exon 68 is not included in the mature mRNA, the single base pair deletion in exon 69 will cause a frameshift and mCherry fusion protein will not be expressed (Figure 2(a)). This minigene was then used as the reporter to screen splicing factors that regulate the alternative splicing of *Cdh23* exon 68.

COS7 cells were transfected with the *Cdh23* minigene reporter and vectors that express different candidate splicing factors fused with GFP. Among all the splicing factors tested, RBM24 and its homolog RBM38 result in robust mCherry fluorescence (Figure 2(b)). Quantification of the red fluorescence using ImageJ shows significant increases in RBM24- or RBM38-exressing cells, but not in cells expressing other splicing factors (Figure 2(c)). This result suggests that RBM24 and RBM38 are able to promote *Cdh23* exon 68 inclusion.

### 3.2. RBM24 and RBM38 Enhance the Inclusion of Cdh23 Exon 68

RT-PCR was then performed to confirm the screening result. The *Cdh23* minigene was expressed in COS7 cells together with RBM24, RBM38, ESRP1, or an empty vector. RT-PCR reveals that exon 68 inclusion is not detectable in mature *Cdh23* mRNA in control cells transfected with an empty vector or ESRP1. However, exon 68 inclusion is significantly enhanced by either RBM24 or RBM38 overexpression (Figure 2(d)).

Small interfering RNAs (siRNAs) were then used to knock down the expression of endogenous *Rbm24* in COS7 cells. All three siRNAs tested inhibit *Rbm24* expression efficiently (Figures 2(e) and 2(f), data not shown). Without overexpressing the splicing enhancers, exon 68 inclusion from *Cdh23* minigene is too weak to be detected with regular RT-PCR (Figure 2(d)), possibly because of the relatively low expression level of endogenous splicing enhancers in COS7 cells. We then used primers specific for *Cdh23(+68)* isoform to examine the inclusion of exon 68. RT-PCR and qPCR results show that *Cdh23(+68)* expression level is significantly decreased by *Rbm24* knockdown (Figures 2(e) and 2(f)). Taken together, both overexpression and knockdown experiments confirm that RBM24/RBM38 could regulate the alternative splicing of *Cdh23* exon 68.

**Figure 3:**
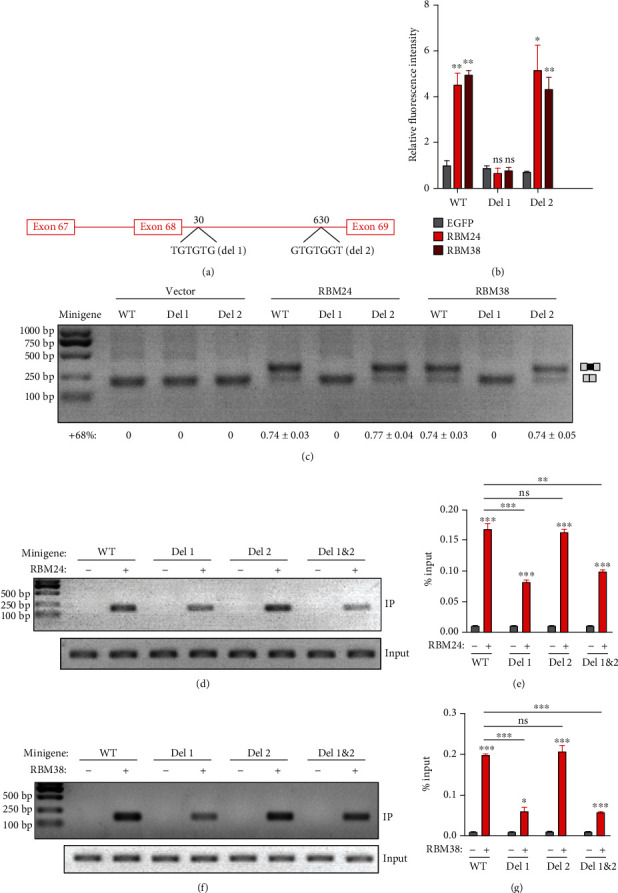
An ISE is required for RBM24-/RBM38-mediated *Cdh23* exon 68 inclusion. (a) Schematic drawing of the locations of the two potential RBM24/RBM38-binding ISEs. (b) Wild-type *Cdh23* minigene or mutants with deletion of the potential RBM24/RBM38-binding ISE were expressed together with RBM24 or RBM38 in COS7 cells. The mCherry fluorescence was examined using a confocal microscope and analyzed using ImageJ software. (c) Wild-type *Cdh23* minigene or mutants with deletion of the potential RBM24/RBM38-binding ISE was expressed together with RBM24 or RBM38 in COS7 cells, and the inclusion of exon 68 in the mature *Cdh23* mRNA was examined by performing RT-PCR. (d–g) Wild-type *Cdh23* minigene or mutants with deletion of the potential RBM24/RBM38-binding ISE were expressed together with RBM24 or RBM38 in COS7 cells. Binding of RBM24/RBM38 with *Cdh23* pre-mRNA was examined by performing native RIP followed by RT-PCR (d, f) or qPCR (e, g). Error bars represent mean ± SEM from triplicate experiments. ^∗^*p* < 0.05, ^∗∗^*p* < 0.01, and ^∗∗∗^*p* < 0.001.

RBM24 and RBM38 have been shown to regulate alternative splicing through binding to a GUGUG motif in the ISEs [23, 24]. There are two potential GTGTG motifs located in intron 68 of the *Cdh23* gene. Motif 1 (TGTGTG) is located 30 bp downstream of *Cdh23* exon 68, while motif 2 (GTGTGGT) is located 630 bp downstream of exon 68 (Figure 3(a)). We examined whether these motifs are involved in the alternative splicing of *Cdh23* exon 68 through deleting either of them in the minigene. Both fluorescence assay and RT-PCR results show that exon 68 inclusion enhancement by RBM24/RBM38 is completely abolished by the deletion of motif 1 but not motif 2 (Figures 3(b) and 3(c)), indicating that motif 1 is required for RBM24/RBM38-regulated inclusion of *Cdh23* exon 68.

RNA immunoprecipitation (RIP) was then performed to examine the physical interaction of RBM24/RBM38 with *Cdh23* pre-mRNA. RT-PCR results show that *Cdh23* pre-mRNA is readily coimmunoprecipitated with RBM24 or RBM38, which is significantly reduced by deletion of motif 1 but not motif 2 (Figures 3(d) and 3(f)). Similar results were obtained by performing qPCR (Figures 3(e) and 3(g)). Taken together, our present data suggest that RNA-binding proteins RBM24 and RBM38 enhance the inclusion of *Cdh23* exon 68 through binding to an ISE motif (UGUGUG).

### 3.3. PTBP1 Inhibits the Inclusion of Cdh23 Exon 68

**Figure 4:**
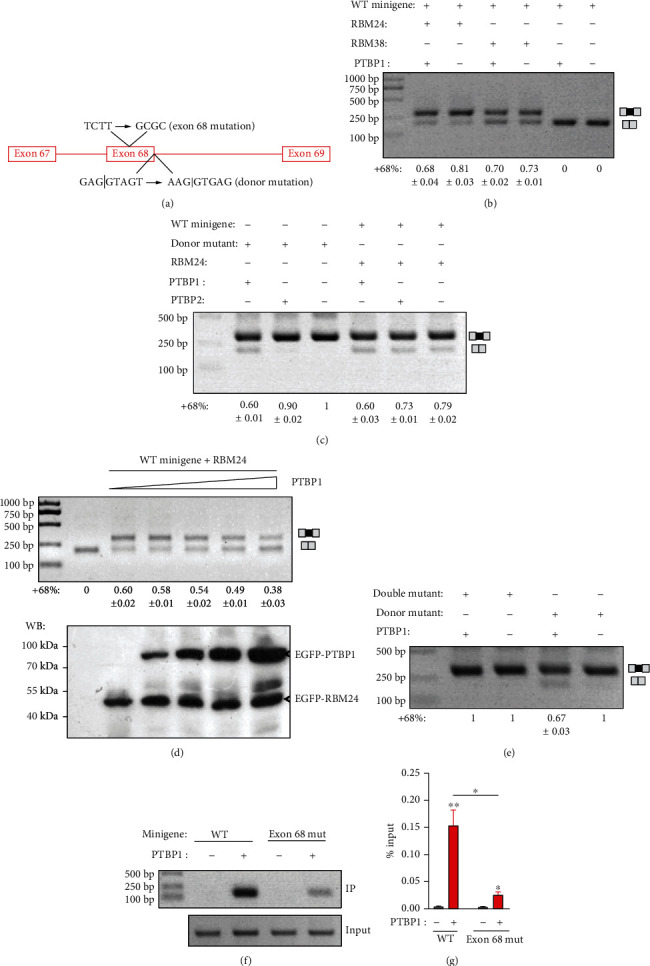
An ESS is required for PTBP1-mediated inhibition of *Cdh23* exon 68 inclusion. (a) Schematic drawing of the location of the potential PTBP1-binding ESS and the donor splicing site of intron 68. (b) *Cdh23* minigene was expressed in COS7 cells together with the splicing regulators as indicated, and the inclusion of *Cdh23* exon 68 was examined by performing RT-PCR. (c) Wild-type *Cdh23* minigene or minigene with donor site mutation was expressed in COS7 cells together with the splicing regulators as indicated, and the inclusion of *Cdh23* exon 68 was examined by performing RT-PCR. (d) *Cdh23* minigene was expressed in COS7 cells together with constant amount of RBM24 and various amount of PTBP1, and the inclusion of *Cdh23* exon 68 was examined by performing RT-PCR. The expression level of RBM24 and PTBP1 was confirmed by performing western blot. (e) *Cdh23* minigene with donor site mutation or donor site/ESS double mutations was expressed in COS7 cells together with PTBP1, and the inclusion of *Cdh23* exon 68 was examined by performing RT-PCR. (f, g) Wild-type *Cdh23* minigene or mutant with deletion of the potential PTBP1-binding ESS was expressed in COS7 cells with or without PTBP1. Binding of PTBP1 with *Cdh23* pre-mRNA was examined by performing native RIP followed by RT-PCR (f) or qPCR (g). Error bars represent mean ± SEM from triplicate experiments. ^∗^*p* < 0.05; ^∗∗^*p* < 0.01.

Next, we want to know how ubiquitously expressed RBM24/RBM38 drives tissue-specific alternative splicing of *Cdh23* exon 68. It has been suggested that most alternative-spliced exons are under combinatorial regulation by both splicing enhancers and repressors [25]. Therefore, RBM24-/RBM38-mediated inclusion of exon 68 in some tissues might be counteracted by yet unknown splicing repressor(s). Bioinformatics analysis suggests a potential ESS motif TCTT in *Cdh23* exon 68, which might be recognized by splicing suppressor PTBP1 (Figure 4(a)) [26]. Consistently, when overexpressed in COS7 cells, PTBP1 inhibits RBM24- or RBM38-mediated inclusion of exon 68, albeit the inhibitory effect is not very robust (Figure 4(b)). We then mutated the weak splice donor site located at the exon 68/intron 68 boundary into an artificial strong donor site, which leads to constitutive exon 68 inclusion (Figure 4(c)). When *Cdh23* minigene with this donor site mutation is used, more robust inhibition of exon 68 inclusion by PTBP1 is observed (Figure 4(c)). PTBP2 is a homolog of PTBP1 that recognizes similar binding motifs [26], and RT-PCR results reveal that both *Ptbp1* and *Ptbp2* transcripts are expressed in the inner ear (Figure 1(b)). However, our present data suggest that PTBP2 inhibits *Cdh23* exon 68 inclusion to a much lesser extent if any compared with PTBP1 (Figure 4(c)).

To mimic the various PTBP1 expression levels in different tissues, we overexpressed PTBP1 in COS7 cells in an increasing gradient. The results show that RBM24-mediated exon 68 inclusion is inhibited by PTBP1 in a dosage-dependent manner (Figure 4(d)). Lastly, we mutated the potential PTBP1-binding site TCTT in exon 68 to GCGC in the presence of the donor site mutation. The results show that the TCTT to GCGC mutation abolishes the inhibitory effect of PTBP1 on exon 68 inclusion (Figure 4(e)). Consistently, the TCTT to GCGC mutation also significantly reduces the binding of *Cdh23* pre-mRNA to PTBP1 revealed by RIP experiment (Figures 4(f) and 4(g)). Taken together, our present data suggest that PTBP1 inhibits *Cdh23* exon 68 inclusion through binding to an ESS motif (UCUU).

### 3.4. RBM24 Regulates the Alternative Splicing of Endogenous Cdh23 Exon 68

**Figure 5:**
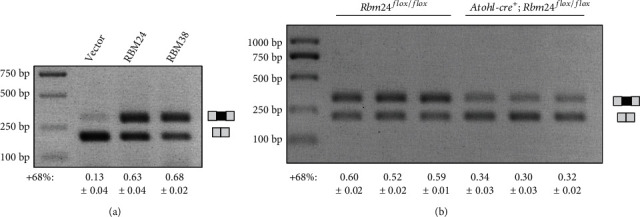
RBM24 regulates the alternative splicing of endogenous *Cdh23* pre-mRNA. (a) RBM24 or RBM38 was overexpressed in HEI-OC-1 cells, and exon 68 inclusion of endogenous *Cdh23* mRNA was examined by performing RT-PCR. (b) RT-PCR was performed to examine *Cdh23* exon 68 inclusion in the inner ear of P2 *Atoh1-cre*; *Rbm24^flox/flox^* (*n* = 3) or *Rbm24^flox/flox^* mice (*n* = 3).

The results discussed above are all obtained using the artificially constructed *Cdh23* minigene. We then used the inner ear cell line HEI-OC-1 to investigate the alternative splicing of endogenous *Cdh23* mRNA. RT-PCR results show that *Cdh23(-68)* is the main splicing isoform expressed in HEI-OC-1 (Figure 5(a)). When RBM24 or RBM38 is overexpressed, *Cdh23(+68)* expression level is significantly increased, suggesting that RBM24 and RBM38 could enhance endogenous *Cdh23* exon68 inclusion (Figure 5(a)).

Lastly, RBM24-mediated alternative splicing of *Cdh23* exon 68 in the inner ear was examined using *Rbm24* knockout mice. *Rbm24* conventional knockout mice die between embryonic day 12.5 (E12.5) and E14.5 because of cardiac problems [24]. In the present work, we used *Rbm24* conditional knockout (cko) mice that disrupt *Rbm24* gene expression in the hair cells. To do so, *Rbm24^loxp/loxp^* mice are crossed with *Atoh1-cre* mice that express Cre recombinase in the developing cochlear hair cells from E14.5 [27]. RT-PCR results show that *Cdh23* exon 68 inclusion is significantly decreased in the inner ear of *Rbm24* cko mice compared with that of control mice (Figure 5(b)), suggesting that RBM24 is required for *Cdh23* exon 68 inclusion *in vivo*.

## 4. Discussion

*Cdh23* exon 68 is subjected to alternative splicing, and *Cdh23(+68)* is preferentially expressed in the inner ear [8, 11–13]. The underlying mechanism for this inner ear-specific alternative splicing of *Cdh23* exon 68 remained unknown. In the present work, we show that the alternative splicing of *Cdh23* exon 68 is regulated by RBM24, RBM38, and PTBP1. RBM24 and RBM38 promote the inclusion of *Cdh23* exon 68, whereas PTBP1 inhibits it.

Several lines of evidences support our conclusion. First, overexpression of RBM24 or RBM38 enhances exon 68 inclusion of the *Cdh23* minigene, while overexpression of PTBP1 inhibits it. Second, mutations of the potential binding sites of RBM24, RBM38, or PTBP1 in the *Cdh23* minigene affect exon 68 inclusion. Third, RIP experiments show that RBM24, RBM38, and PTBP1 bind *Cdh23* pre-mRNA, which is affected by mutations of their potential binding sites. Fourth, knockdown of *Rbm24* expression with siRNAs decreases exon 68 inclusion of the *Cdh23* minigene. Fifth, overexpression of RBM24 or RBM38 promotes exon 68 inclusion of endogenous *Cdh23* in HEI-OC-1 cells. Last, inclusion of *Cdh23* exon 68 is reduced in the inner ear of *Rbm24* cko mice.

There are two potential RBM24/RBM38-binding ISEs downstream of exon 68, and our data suggest that mutation of site 1 but not site 2 affects the alternative splicing of *Cdh23* exon 68. Alignments of genomic sequences flanking *Cdh23* exon 68 among different mammals show that site 1 is evolutionally conserved while site 2 is not (data not shown), consistent with an important role of site 1 in mediating *Cdh23* exon 68 inclusion. There is also a potential PTBP1-binding ESS located in *Cdh23* exon 68, and mutation of this site affects the inhibition of *Cdh23* exon 68 inclusion by PTBP1. Interestingly, the PTBP1 homolog PTBP2 shows a much weaker inhibitory effect on *Cdh23* exon 68 inclusion, albeit it is expressed in the inner ear and recognizes similar binding motifs.

A question that remained is how relatively ubiquitously expressed RBM24, RBM38, and PTBP1 regulate inner ear-specific alternative splicing of *Cdh23* exon 68. Alternative splicing has been proposed to be regulated by the combinatorial action of splicing enhancers and repressors [25]. Transcriptome RNAseq reveals high expression level of *Rbm24* and low expression level of *Rbm38* and *Ptbp1* in the auditory and vestibular hair cells, which is consistent with the inner ear-specific *Cdh23* exon 68 splicing [28]. Additionally, there might be other splicing factors that regulate the alternative splicing of *Cdh23* exon 68 but are not included in our screening in the present work. In line with this, *Cdh23* exon 68 inclusion is decreased but still present in the inner ear of *Rbm24* cko mice. qPCR reveals that *Rbm38* expression remains on the similar low level in the inner ear of *Rbm24* cko mice (data not shown), suggesting that splicing enhancer other than RBM24 and RBM38 might be responsible for the remnant inclusion of *Cdh23* exon 68 in the *Rbm24* cko mice.

At present, only a few splicing factors have been identified to play important roles in the inner ear, including SRRM4, SFSWAP, and ESRP1 [18–20]. Our present work adds RBM24 to this growing list. RBM24 is an RNA-binding protein that contains an RNA-recognition motif (RRM) and an alanine-rich low-complexity region. It has been shown that RBM24 regulates pre-mRNA alternative splicing as well as mRNA stability and translation [24, 29–38]. RBM24 is a major regulator of muscle-specific alternative splicing, and global *Rbm24* inactivation affects cardiac development that eventually leads to embryonic death [24]. In the mouse inner ear, RBM24 expression is specifically detected in the hair cells, but its physiological role in the inner ear remains unknown [22, 39]. Here, we show that RBM24 regulates the alternative splicing of *Cdh23*; hence, it might play an important role in the development and/or function of hair cells. Meanwhile, immunostaining shows that RBM24 is also localized in the hair cell stereocilia, suggesting that it might also play some roles in the organization and/or function of this F-actin-based cell protrusion [22]. Super-resolution microscopy [40–42] and conditional knockout mice will certainly help to learn more about the role of RBM24 in the inner ear.

## Data Availability

The data supporting the findings of this study are available within the article.
